# Effects of Tamarind (*Tamarindus indica* L.) Pulp-Based Marination on Physicochemical Quality, Oxidative Stability, Microbiological Characteristics, and Sensory Attributes of Eye of Round Beef

**DOI:** 10.3390/foods15162783

**Published:** 2026-08-08

**Authors:** Brenno Guimarães Barreto, Juliana Sant’Ana Falcão Leite, Camila Cristina Avelar de Souza, Hélio de Santana Silva, Raick Alves Ribeiro, Ronaldo Lopes Oliveira, Maurício Costa Alves da Silva, Ana Maria Herrero, Claudia Ruiz-Capillas, Carlos Pasqualin Cavalheiro

**Affiliations:** 1 Laboratory of Meat and Meat Products Industry and Inspection (LabCarne), School of Veterinary Medicine and Animal Science, Federal University of Bahia, Salvador 40170-115, Bahia, Brazil; brenno.guimaraes@ufba.br (B.G.B.); juliana.falcao@ufba.br (J.S.F.L.); camila.cristina@ufba.br (C.C.A.d.S.); helio.silv4@gmail.com (H.d.S.S.); mcasilva@ufba.br (M.C.A.d.S.); 2 Graduate Program in Food Science, Faculty of Pharmacy, Federal University of Bahia, Salvador 40170-010, Bahia, Brazil; ribeiroraick@gmail.com (R.A.R.); ronaldooliveira@ufba.br (R.L.O.); 3 INDMEAT Group, Department of Meat and Fish Products, Institute of Food Science, Technology and Nutrition (ICTAN-CSIC), 28040 Madrid, Spain; ana.herrero@ictan.csic.es; 4 Graduate Program in Animal Science in the Tropics, School of Veterinary Medicine and Animal Science, Federal University of Bahia, Salvador 40170-010, Bahia, Brazil

**Keywords:** beef marination, clean-label meat products, sensory analysis, color parameters, oxidative stability

## Abstract

This study evaluated the effects of tamarind (*Tamarindus indica* L.) pulp as a marinade on the physicochemical quality (marinade liquid uptake, water-holding capacity, cooking loss, proximate composition, water activity, pH, color, and texture), oxidative stability (lipid oxidation), microbiological characteristics (mesophilic aerobic counts, *Enterobacteriaceae,* Gram-positive cocci, and molds and yeasts), and sensory attributes of eye of round beef (*M. semitendinosus*). Three treatments were compared: non-marinated control (C), brine (B; 2.5% NaCl), and NaCl with tamarind pulp (BPT; 2.5% NaCl + tamarind pulp). Tamarind incorporation significantly reduced pH and water-holding capacity, resulting in lower moisture content and higher ash and carbohydrate levels. BPT samples exhibited increased lightness (L*) and yellowness (b*) and reduced redness (a*), reflecting pigment contribution and acid-induced structural modifications. Texture profile analysis showed lower hardness and chewiness in BPT throughout storage, demonstrating improved tenderness. Lipid oxidation increased in B but remained controlled in BPT, highlighting the antioxidant potential of tamarind pulp. Tamarind selectively improved the microbiological profile by inhibiting *Enterobacteriaceae* and reducing mesophilic aerobic bacteria, although it was less effective in controlling molds and yeasts. Sensory evaluation showed that B treatment received the highest scores for flavor and overall acceptability, while BPT was characterized by fruity, acidic, and sweet notes, resulting in a distinctive sensory profile without reducing acceptability relative to the control. Overall, tamarind pulp substantially modulated the quality characteristics of marinated eye of round beef, providing advantages in oxidative stability, microbial control, and texture while also reducing water-holding capacity, altering color, increasing mold and yeast growth, and producing a distinctive sensory profile. These findings highlight both the potential and the limitations of tamarind pulp as a natural ingredient for clean-label meat products.

## 1. Introduction

Beef marination is widely used to improve the technological and sensory quality of meat, particularly in cuts that are low in tenderness and have reduced consumer acceptability. Among beef muscles, the eye of round is commonly associated with low tenderness, reduced juiciness, and less desirable flavor profiles, which reduce its commercial value and consumer preference [1,2]. In this context, marination has become an important technological strategy for enhancing tenderness, flavor, juiciness, and overall eating quality [3], while also contributing to the valorization of lower-value meat cuts.

In recent years, increasing consumer demand for clean-label meat products has stimulated interest in replacing synthetic additives with natural ingredients with antioxidant and antimicrobial properties. Within this context, the incorporation of fruit-based ingredients into marinades has emerged as a promising approach, owing to natural abundance, compositional diversity, and functional properties. Fruits are rich sources of organic acids, pigments, and bioactive compounds, which may modify meat physicochemical characteristics and microbial stability. Previous studies have demonstrated that fruits such as lemon, pineapple, berries, blackcurrant, and papaya may improve meat tenderness and oxidative stability through acidification, proteolytic activity, and antioxidant effects [3,4]. Although fruit-derived ingredients have attracted considerable attention as clean-label alternatives, their successful industrial implementation depends on consistent ingredient composition, formulation standardization, microbiological stability, and consumer acceptance. Therefore, evaluating both the benefits and limitations of these ingredients is essential before practical application in meat processing. Additionally, the effects of fruit-based marinades are highly dependent on fruit composition, acidity, enzyme activity, antioxidant activity, and interactions with muscle proteins, with consequences to technological, shelf life, and sensory aspects [5,6].

The marination of beef has become an increasingly adopted strategy to enhance sensory attributes such as flavor, tenderness, and color [7,8]. This growing interest is closely aligned with the expansion of the ready-to-cook meat segment, driven by consumer demand for convenience and improved eating quality [8]. However, the effectiveness of marination is highly dependent on the composition of the marinade and its interaction with the muscle structure, underscoring the need for novel formulations that deliver consistent technological and sensory improvements.

Among tropical fruits, tamarind (*Tamarindus indica* L.) has attracted increasing attention due to its chemical composition. Tamarind pulp is characterized by significant amounts of bioactive compounds, such as total phenolics, flavonoids, and vitamin C. In addition, its antioxidant activity has been demonstrated through DPPH and ABTS assays [5,9]. These attributes suggest the potential to influence meat quality through multiple pathways, including pH-mediated protein modification, lipid oxidation inhibition, and color stabilization. Although tamarind has been previously reported as a natural tenderizing agent [10], its application as a marinade liquid in meat remains insufficiently explored [11,12].

Although fruit-based marinades have been increasingly investigated, limited information is available on the application of tamarind pulp to structurally dense, low-juiciness, less desirable flavor profiles, and low-value beef cuts, such as eye of round beef, during refrigerated storage. Therefore, we hypothesized that tamarind pulp could act as a multifunctional natural marination ingredient, improving oxidative stability and tenderness while modulating the microbial and sensory properties of eye of round beef. Thus, the aim of this study was to evaluate the effects of tamarind pulp-based marination on the physicochemical quality, oxidative stability, microbiological characteristics, and sensory attributes of eye of round beef.

## 2. Materials and Methods

### 2.1. Raw Material

Eye of round beef (*M. semitendinosus*) was obtained from six different animals of the Nellore breed (2–3 years old, approximately 300 kg; 74% moisture, 20% protein, 3% fat, 2% ash, and pH 5.63), purchased one day after slaughter from a local meat supplier (Salvador, Brazil). The excessive fat and conjunctive tissue were removed, and the eye of round beef was cut into 1.5 cm thickness and 125 ± 10 g slices. Slices obtained from each animal were randomly allocated among the three experimental treatments (C, B, and BPT), so that each animal contributed samples to all treatments. Frozen pasteurized tamarind pulp (8% carbohydrates, 6% total sugars, 1.5% fiber, pH 2.50, and color parameters: L*: 38.3, a*: 9.8, and b*: 19.2; Polpas Pedra Branca, Salvador, Brazil) was purchased from a local market. Sodium chloride (NaCl; Sciavicco, Belo Horizonte, Minas Gerais, Brazil) was used to prepare the brine solution.

### 2.2. Eye of Round Beef Preparation and Marination

Three experimental treatments were prepared in duplicate: unmarinated control (C), brine-marinated (B), and tamarind pulp-marinated (BPT). The brine used for the B treatment consisted of 2.5% (*w*/*v*) NaCl dissolved in distilled water. The BPT treatment used undiluted tamarind pulp as a marinade liquid, and NaCl was added at 2.5% (*w*/*v*), calculated based on the total volume of tamarind pulp. A total of 90 slices (1.5 cm thick; 125 ± 10 g) were prepared, corresponding to five slices per treatment × three treatments (C, B, BPT) × three sampling times (0, 3, and 7 days) × two replications. Eye of round beef samples were completely immersed in the respective marinades at a 1:1 (*v*/*w*) marinade–to-meat ratio in high-density polyethylene trays lined with plastic film and stored at 4 °C for 20 h. Subsequently, the samples were drained for 5 min, vacuum-packed (Registron, São Paulo, Brazil), and stored at 4 °C for 7 days. In this context, technological properties, proximate composition, and sensory analysis were performed after the marination process, while water activity, pH, lipid oxidation, instrumental color, instrumental texture, and microbiological analysis were performed during storage.

### 2.3. Technological Properties

Eye of round beef (three per treatment) was weighed before and after the marination process, and the percentage of marinade liquid uptake (MLU) was calculated as follows [13]:

$$MLU\;\left(\%\right)=\;\left(\frac{Marinated\;weight-Non\;marinated\;weight}{Non\;marinated\;weight}\right)\;\times \;100$$

The water-holding capacity (WHC) was determined based on the technique of Hamm [14], as described by Rupasinghe et al. [15]. Eye of round beef samples (3 g) were placed between two sheets of double-ring filter paper (No. 1; Whatman™, Cytiva, Shanghai, China) and pressed between two acrylic plates using a 5 kg weight for 5 min. Then, the samples were weighed again, and the WHC was calculated as follows:

$$WHC\;\left(\%\right)=\;100-\left(\left(\frac{Initial\;weight-Final\;weight}{Initial\;weight}\right)\;\times \;100\right)$$

Eye of round beef samples were weighed and cooked for 9 min on a preheated electric grill (PGR03P, Philco, São Paulo, Brazil) at 160 °C until the internal temperature reached 72 °C. Samples were turned every minute to ensure uniform cooking. After cooling to room temperature (25 °C), they were gently blotted with a paper towel to remove excess surface water and reweighed. The cooking loss (CL) was calculated as follows [16]:

$$CL\;\left(\%\right)=\;\left(\frac{Raw\;weight-Cooked\;weight}{Raw\;weight}\right)\;\times \;100$$

### 2.4. Proximate Composition

Proximate composition was determined in triplicate using a FoodScan™ 2 Lab TS analyzer (FOSS Analytical, Hillerød, Denmark) for moisture, protein, fat, ash, carbohydrate, and salt content [17].

### 2.5. Water Activity (a_w_)

Water activity was measured at 25 °C using a Nov-Labtouch C/Cm-2 device (Novasina, Lachen, Switzerland) in triplicate during storage [18].

### 2.6. pH

In a food processor (RI7630, Phillips Walita, São Paulo, Brazil), 10 g of each sample was homogenized in 90 mL of distilled water for 1 min at 25 °C. Then, the pH was measured with a calibrated pH meter (mPA-210; Tecnopon, São Paulo, Brazil) using standard buffer solutions (pH 4.00 and 7.00 at 25 °C). Measurements were performed in quintuplicate during storage.

### 2.7. Lipid Oxidation

Lipid oxidation was assessed using the thiobarbituric acid reactive substances (TBARS) assay described by Triki et al. [19] with slight modifications. Five grams of each sample were homogenized with 35 mL of 7.5% trichloroacetic acid (Êxodo Científica, São Paulo, Brazil) for 30 s. The mixture was filtered, and 5 mL of filtrate was collected and mixed with 5 mL of 20 mM thiobarbituric acid (Êxodo Científica, São Paulo, Brazil). The resulting solution was kept in the dark at 20 °C for 20 h. The color formed in the solution was determined using a spectrophotometer (Metash Instruments Co., Ltd., Shanghai, China) at 532 nm. The calibration curve was plotted with 1,1,3,3-teraethoxypropane (Sigma, Steinheim, Germany), and results were expressed as mg malondialdehyde (MDA)/kg sample.

### 2.8. Instrumental Color

Color was measured in quintuplicate using a colorimeter (NR200, 3nh, Shenzhen, China) with illuminant D-65, a 10° standard observer, and an 8 mm aperture. Eye of round beef samples were removed from the packaging and exposed to atmospheric air for 10 min prior to color measurement [20]. Lightness (L*), redness (a*), yellowness (b*), chroma (C*), and hue angle (*h*°) parameters were measured on the surface of samples.

### 2.9. Texture Profile Analysis (TPA)

Texture profile analysis was performed on cooked eye of round beef samples using a texture analyzer (TA.XT Express, Stable Micro Systems Ltd., Surrey, UK). Two compression cycles with a 5 s interval were applied using an SMS P/0.5 probe and a 5 kg load cell. Pre-test speed was 3.0 mm/s, test speed 1.0 mm/s, post-test speed 3.0 mm/s, and trigger force 10 g. Hardness (N), chewiness (N × mm), cohesiveness, and springiness (mm) were recorded.

### 2.10. Microbiological Analysis

A 10 g sample of each raw eye of round beef was aseptically transferred to a stomacher bag and homogenized with 90 mL of sterile 0.1% peptone water (Kasvi, São José dos Pinhais, Brazil) for 30 s in a stomacher (LS Logen 1901, Logen Scientific, São Paulo, Brazil). Plate Count Agar (BD Difco, A Ponte Claix, France) was used for total mesophilic aerobic counts (48 h at 35 °C). Violet Red Bile Lactose Agar (Kasvi, São José dos Pinhais, Brazil) was used for enumeration of *Enterobacteriaceae* (24 h at 35 °C). Acidified Potato Dextrose Agar (Himedia, Mumbai, India) was used for enumeration of molds and yeasts (5 days at 25 °C). The presence or absence of *Salmonella* spp. was determined in 25 g of each sample.

### 2.11. Sensory Analysis

Sensory evaluation was conducted one day after marination. A 10 cm structured line scale, anchored at “Dislike extremely” and “Like extremely”, was used to assess appearance, flavor, color, texture, odor, and overall acceptability. Purchase intention was assessed with a 6 cm scale anchored with “Definitely would not buy” and “Definitely would buy”. A total of seventy consumers (61.42% female, 38.57% male, aged 19–55 years) participated, evaluating 210 samples (70 panelists × 3 treatments). Additionally, a check-all-that-apply (CATA) questionnaire with 24 descriptors was used. Samples were cooked for 9 min on an electric grill (PGR03P, Philco, São Paulo, Brazil) until reaching 72 °C, cut into 2 × 2 × 1 cm pieces (~20 g), and served in coded disposable containers. Water and unsalted crackers were provided for palate cleansing between samples.

### 2.12. Statistical Analysis

The experiment was conducted in two experimental repetitions. Each beef slice was considered one experimental unit. Since analyses were performed in triplicate, the three determinations represented technical replicates of the same experimental unit, and their mean value was used for statistical analysis. Analysis of variance (ANOVA) was carried out using SPSS 25.0 (SPSS Inc., Chicago, IL, USA) to evaluate the effects of marination and storage time on the technological, physicochemical, microbiological, and sensory properties of eye of round beef. A completely randomized design was applied, with treatments (C, B, BPT) and storage times (0, 3, 7 days) as fixed effects, and replications as random effects. Interactions between fixed effects were also considered in the analyses performed during the storage period. Means were compared using Tukey’s HSD test, and the differences were considered significant when *p* < 0.05. For the CATA data, the frequency of mention for each attribute was determined, and Cochran’s Q test was applied to detect differences among treatments. Correspondence analysis was used to explore associations between categorical variables.

## 3. Results and Discussion

### 3.1. Technological Properties

The technological properties of marinated eye of round beef are presented in Table 1. The results indicate that the incorporation of tamarind pulp into the brine system markedly influenced the technological behavior of the meat, highlighting the complex interplay between ionic strength and acid-induced protein modifications. The MLU values ranged from −1.66% to 9.27% (Table 1), with the lowest value observed in the C treatment, as expected due to moisture loss during refrigerated storage in the absence of protective marinade. Both brine and tamarind pulp significantly affected MLU (*p* < 0.05). The highest uptake observed in the brine-treated samples (B) is consistent with the salting-in effect of sodium chloride, which enhances myofibrillar protein solubilization and promotes water binding, thereby facilitating marinade diffusion into the muscle matrix [21]. In contrast, the reduced MLU in the tamarind-containing treatment (BPT) suggests that acidification partially counteracted the effects of salt. This behavior may be explained by pH-induced protein denaturation, reducing net charge and limiting the availability of binding sites for water and solutes. As a result, the structural shrinkage of myofibrils impaired marinade retention, restricting its incorporation into the meat.

A similar pattern was observed for WHC, which was lower (*p* < 0.05) in BPT (74.16%) compared to C (84.26%) and B (82.33%). Although brine marination improved WHC due to increased ionic strength [22], the absence of differences between C and B treatments (*p* > 0.05) suggests that the salting conditions applied may have been insufficient to improve WHC in this relatively dense muscle. Alternatively, the intrinsic characteristics of the eye of round beef may have limited the extent of salt-induced swelling. Lower pH muscle was associated with lower WHC [23]. Then, the reduction in WHC observed in BPT is associated with the denaturation of myofibrillar and sarcoplasmic proteins induced by the low pH of tamarind pulp, which decreases the ability of the muscle to retain immobilized water [24]. Interestingly, the acidic pH of tamarind pulp similarly affected both MLU and WHC (Table 1).

Despite the lower WHC in BPT, CL was not affected by treatment (*p* > 0.05), with values ranging from 34.36% to 37.66% (Table 1). This apparent discrepancy suggests that water losses induced by acidification occurred predominantly during marination rather than during thermal processing. According to Zielbauer et al. [25], the water located between the myofibrils is squeezed out due to the disruption of cell walls and the shrinking of myofibrils. In this context, if the water had already been lost due to marinade acidification, heat-induced protein denaturation during cooking minimized structural differences between treatments, resulting in similar water losses. These findings reinforce the notion that WHC and CL are not always directly correlated and are influenced by distinct physicochemical mechanisms. Although marination is often associated with improved juiciness and reduced water loss during cooking, such effects are dependent on marinade composition and processing conditions. Similar CL values have been reported by Sabzi et al. [21] in beef marinated with unripe grapes.

### 3.2. Proximate Composition

The proximate composition of the eye of round beef samples is presented in Table 1, with all parameters affected by the treatments (*p* < 0.05). Moisture content ranged from 65.65% to 71.26%, with the lowest values observed in the BPT treatment (*p* < 0.05). Although the C treatment exhibited higher moisture content than BPT (*p* < 0.05), this result can be explained by the nature of marinade uptake, which reflects total weight gain rather than water retention alone. The combination of salt and tamarind pulp in BPT created a hypertonic, acidic environment, promoting osmotic gradients that favored water loss from the muscle [26], while simultaneously favoring the uptake of solutes such as sugars and minerals. This interpretation is supported by the higher ash and carbohydrate contents observed in BPT (*p* < 0.05; Table 1). Comparable reductions in moisture have been reported in pork loin marinated with sour cherry and plum juices [24], whereas the opposite trend was observed in Boston butt pork marinated with yellow mombin pulp [3], highlighting the influence of marinade composition on water dynamics.

Protein content exhibited an inverse trend, with the highest value observed in the C treatment (23.96%), followed by the BPT (22.39%) and B (18.97%) treatments (*p* < 0.05). The lower protein content in marinated samples can primarily be attributed to dilution effects and to the leaching of water- and salt-soluble protein into the marinade, particularly under conditions that promote protein solubilization [27,28], such as the 2.5% NaCl concentration used in this study. The relatively higher protein content in BPT compared to B is therefore more closely associated with its reduced moisture content rather than an actual increase in protein retention. In addition, acid-induced protein denaturation in BPT may have reduced protein solubility [27], limiting their diffusion into the medium and thereby attenuating protein losses. Moreover, reduced electrostatic repulsion, as the pH approaches the isoelectric point of meat proteins, further facilitates aggregation [29], potentially contributing to the increased protein levels observed. Conversely, Beltrán-Cotta et al. [3] found lower protein content in Boston butt pork marinated with yellow mombin than in non-marinated samples.

Fat content ranged from 3.59% to 7.22%, with higher values in BPT treatment (*p* < 0.05). This increase is explained by a concentration effect from moisture reduction, rather than by changes in lipid content per se. The highest ash and carbohydrate contents were observed in the BPT treatment (*p* < 0.05) (Table 1), which is consistent with the compositional profile of tamarind pulp. These results indicate that solute incorporation during marination contributed substantially to the observed compositional changes. However, the magnitude of solute uptake is strongly influenced by the specific physicochemical properties of the fruit matrix, particularly its soluble contents and acidity.

As expected, higher salt contents were observed in both marinated treatments, reflecting the use of a 2.5% brine solution. The low intrinsic sodium content of tamarind pulp did not significantly influence salt levels in BPT, resulting in no differences between marinated treatments (*p* > 0.05) (Table 1). The sodium content in the C treatment (0.09%) is consistent with values reported for unprocessed meat [30].

### 3.3. Water Activity (a_w_)

The water activity (a_w_) values of eye of round beef samples during the 7-day storage period are presented in Table 2. Significant effects of treatment, storage time, and their interaction were observed (*p* < 0.05), indicating that both formulation and storage dynamics influenced the physicochemical state of water within the muscle. At the beginning of storage, values ranged from 0.974 to 0.979, and the incorporation of tamarind pulp (BPT) resulted in the lowest a_w_ values, particularly on day 3 (*p* < 0.05). This reduction is most plausibly associated with the high concentrations of soluble solids in tamarind pulp, including organic acids and sugars, which contribute to lowering a_w_. In this context, the decrease in aw reflects not only changes in water distribution but also increased solute-water interactions within the meat matrix. Although the acidic nature of tamarind pulp may have contributed by promoting protein denaturation and structural changes, its effect on aw is secondary compared to the influence of dissolved solutes. The lower WHC observed in BPT further supports the reduced a_w_ results (Table 2). Comparable decreases in a_w_ have been reported in pork marinated with solute-rich matrices, such as fermented dairy products [31], supporting the role of compositional factors in modulating a_w_. However, despite these differences, all a_w_ values remained within the typical range reported for fresh and marinated meat.

### 3.4. pH

The pH values of eye of round beef samples during the 7-day storage period are presented in Table 2. Significant effects of treatment, storage time, and their interaction were observed (*p* < 0.05), indicating that both marinade composition and storage conditions influenced the acid-base equilibrium of the meat. The BPT treatment consistently exhibited lower pH values (4.02–4.24) than the C and B treatments across all storage days (*p* < 0.05), reflecting the incorporation of tamarind pulp, which naturally has a low pH (approximately 2.5).

From a physicochemical perspective, the reduction in pH brought muscle proteins closer to their isoelectric point, where their net electrical charge is minimized. Under these conditions, electrostatic repulsion among protein molecules decreases, promoting closer protein–protein interaction and reducing the intermolecular space available for water retention within the myofibrillar structure [32]. Consequently, WHC is reduced, contributing to the lower moisture content observed in the BPT treatment. In addition to these effects on water retention, the acidic conditions may induce partial structural modifications of myofibrillar proteins and facilitate the thermal conversion of collagen to gelatin during cooking, thereby improving tenderness [21]. Similar reductions in pH have been reported in meat marinated with fruit-derived ingredients, reinforcing the role of organic acids in modulating meat acidification [33].

Storage time also influenced pH values, with a gradual decline observed over the 7-day period across all treatments. This trend may be associated with microbial activity, particularly the growth of acid-producing microorganisms, such as lactic acid bacteria, as well as ongoing biochemical processes, including residual glycolysis and the accumulation of organic acids during refrigerated storage [34].

### 3.5. Lipid Oxidation

Lipid oxidation, as measured by TBARS, is presented in Table 2. Treatment, storage time, and their interaction significantly influenced TBARS values (*p* < 0.05), indicating that both marination and storage dynamics affected oxidative stability. At day 0, the C treatment exhibited the lowest TBARS value (0.145 mg MDA/kg), whereas both marinated samples showed higher values (*p* < 0.05), but with no differences between B and BPT (*p* > 0.05). This pattern suggests that the marination process itself may have promoted initial lipid oxidation. Such an effect is consistent with the pro-oxidant activity of NaCl, which can disrupt cellular membranes, increase lipid exposure to pro-oxidative agents, and inhibit antioxidant enzymes [35]. In addition, mechanical handling and increased oxygen exposure during marination may have further contributed to the lipid oxidation at the beginning of storage. Similar patterns have been reported in Boston butt pork marinated in yellow mombin juice at early storage stages [3].

By the end of the storage period, the B treatment exhibited the highest TBARS value (0.219 mg MDA/kg), which is consistent with the sustained pro-oxidant effect of NaCl during storage. In contrast, the BPT treatment showed TBARS values comparable to the C (*p* > 0.05) and lower than those of the brine treatment (B) (*p* < 0.05), indicating that the incorporation of tamarind pulp mitigated lipid oxidation over time. This protective effect is associated with the presence of phenolic compounds (148.77 mg GAE/100 g), flavonoids (39.99 mg CTE/100 g), vitamin C (201.7 mg/100 g), and carotenoids in tamarind pulp [5], which act as radical scavengers and metal chelators, thereby interrupting the propagation phase of lipid peroxidation [36]. In this context, the antioxidant capacity of tamarind (DPPH: 1144.33 µmol Trolox/100 g and ABTS: 1226.54 µmol Trolox/100 g) [5,9] appears to counterbalance the pro-oxidant effect of NaCl, thereby improving oxidative stability. Although decreases in TBARS values during storage can be attributed to MDA degradation by spoilage microorganisms [37,38], this mechanism is unlikely to fully explain the observed behavior in BPT. Importantly, all TBARS values remained well below the threshold associated with perceivable rancidity [39], with no treatment exceeding 0.30 mg MDA/kg throughout storage.

### 3.6. Instrumental Color

The instrumental color parameters of the eye of round beef samples during the 7-day storage period are presented in Table 3. Both treatment and storage time significantly influenced some color attributes, reflecting the combined effects of marinade composition, meat properties, and oxidative processes. The incorporation of tamarind pulp markedly affected color, resulting in meat that appeared lighter, less red, and more yellow than in the C treatment. This effect is consistent with the intrinsic color of tamarind pulp, which is characterized by brownish-yellow hues and a relatively low redness intensity.

Lightness (L*) was significantly influenced by treatment (*p* < 0.05), whereas storage time and the interaction between storage time and treatment had no effect (*p* > 0.05). Marinated samples exhibited higher L* values than C at early stages of storage. This increase is commonly associated with salt-induced changes in muscle structure, where increased ionic strength promotes protein denaturation and enhances light scattering within the tissue [40]. However, on days three and seven, no differences were found between C and B, while BPT treatment consistently showed higher L* values than C across all storage days (*p* < 0.05). In addition, acidification in BPT promoted protein swelling, contributing to structural modifications that increase surface reflectance [41]. Similar results have been reported in meat marinated with unripe grapes and mushroom powders [21,42].

Redness (a*) is a key indicator of meat freshness and strongly influences consumer acceptance of meat and meat products [43], and it was significantly affected by treatment, storage time, and their interaction (Table 3; *p* < 0.05). The C sample exhibited the highest a* values at early storage stages, whereas marinated treatments showed reduced redness, particularly at day 0 (*p* < 0.05). This reduction may be attributed to the promotion of metmyoglobin formation in the presence of NaCl, which accelerates oxidative processes and contributes to a brownish discoloration [44]. In addition, the increased lipid oxidation observed in marinated samples (Table 2) may have contributed to myoglobin oxidation through secondary oxidation products, reinforcing the decline in redness values. Although lipid oxidation in marinated meat may have influenced redness values, the presence of pigments in the marinade can also affect visual perception. For example, pork marinated with sour cherry showed increased redness due to its high anthocyanin content [24].

Yellowness (b*) was significantly influenced by treatment, storage time, and their interaction (*p* < 0.05), with the highest values observed in the BPT treatment on days 0 and 3 (*p* < 0.05). This behavior is associated with the presence of yellow pigments in tamarind pulp, including carotenoids and flavonoids [5,45]. These pigments may also have contributed to the lower a* values observed in BPT treatment (Table 3). The decline in yellowness in BPT over time (from 14.7 to 11.9) suggests progressive pigment degradation, due to oxidative reactions during storage. In contrast, the more stable b* values observed in C and B treatments indicate the absence of pigment oxidation.

Chroma (C*) and hue angle (*h*°) provided additional insight into color stability. Chroma, a measure of color intensity, decreased over storage time across treatments, indicating progressive discoloration (*p* < 0.05). The lowest C* values were observed in the B treatment at day 0 (*p* < 0.05), indicating the pro-oxidant effect of NaCl at early stages of storage. Hue angle was significantly affected by treatment, with higher values observed in BPT, consistent with a shift toward yellowish-brown tones and reduced redness. In general, these changes indicate myoglobin oxidation and pigment interactions within the meat matrix.

### 3.7. Texture Profile Analysis

**Figure 1:**
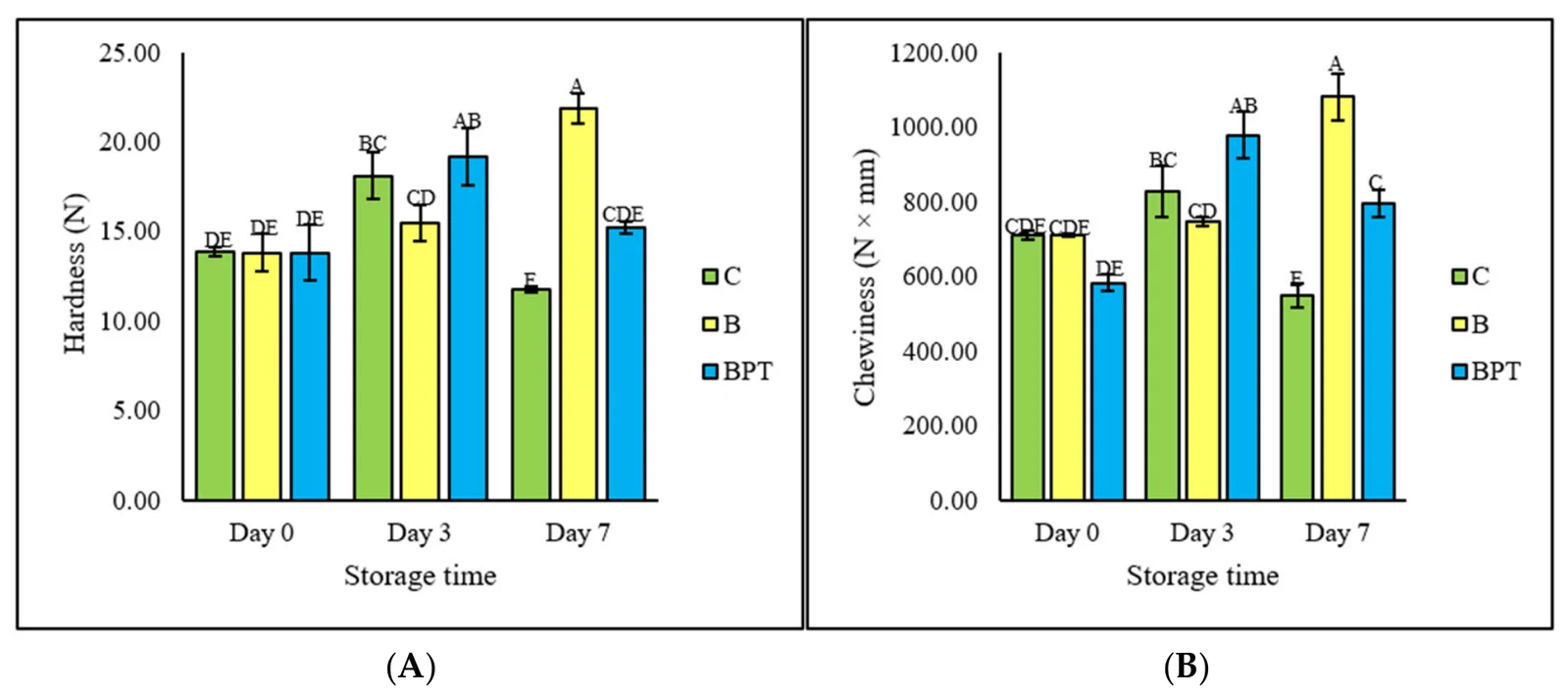
Hardness (**A**) and chewiness (**B**) values of cooked eye of round beef. Different uppercase superscripts (A–E) indicate significant differences (*p* < 0.05). C: Unmarinated samples; B: Brine-marinated samples; BPT: Tamarind-brine marinated samples. Error bars represent the standard error of the mean.

Texture is a key quality attribute influencing consumer perception of meat products. The results of the texture profile analysis for eye of round beef samples during storage are presented in Figure 1. Hardness and chewiness were significantly affected by treatment, storage time, and their interaction (*p* < 0.05), whereas cohesiveness and springiness remained unchanged (*p* > 0.05), suggesting that the applied treatments primarily influenced resistance to deformation rather than structural recovery properties.

At the beginning of storage, no differences in hardness were observed among the treatments (*p* > 0.05). However, hardness increased from day 0 to day 3, indicating structural changes occurring during early storage. From day 3 to day 7, this increase persisted only in the brine-treated samples (B), whereas hardness declined in both control (C) and tamarind-treated samples (BPT). The initial increase in hardness is associated with moisture redistribution and partial water loss, particularly in the C treatment, as well as with structural modifications induced by NaCl in marinated samples. Although moderate salt concentrations generally promote protein solubilization (salting-in) [46], prolonged storage may promote protein aggregation and structural changes that increase resistance to compression. In contrast, the reduction in hardness observed in C and BPT during the later storage stage may be attributed to distinct mechanisms. In the C treatment, the decrease in hardness is consistent with the aging process, in which endogenous proteolytic enzymes such as calpains, cathepsins, caspases, and proteasomes degrade myofibrillar proteins, thereby enhancing meat tenderness [47]. In BPT, the acidic environment created by tamarind pulp contributed to structural weakening through combined physicochemical and biochemical effects. Acid-induced protein denaturation and reduced electrostatic repulsion may promote myofibrillar contraction, while also increasing susceptibility to proteolytic activity. In addition, the presence of organic acids may facilitate the solubilization of connective tissue components, further contributing to texture softening [10,48].

A similar pattern was observed for chewiness, which is directly influenced by hardness. While no differences were detected at the beginning of storage (*p* > 0.05), the B treatment exhibited higher chewiness values at the end of storage (*p* < 0.05) (Figure 1). The lower hardness and chewiness observed in BPT can be considered technologically advantageous, as they indicate improved tenderness. These findings are consistent with previous studies reporting that acidic fruit-based marinades reduce texture parameters in meat [3,21,28], reinforcing the potential of tamarind pulp as a natural tenderizing agent [10].

### 3.8. Microbiological Analysis

**Figure 2:**
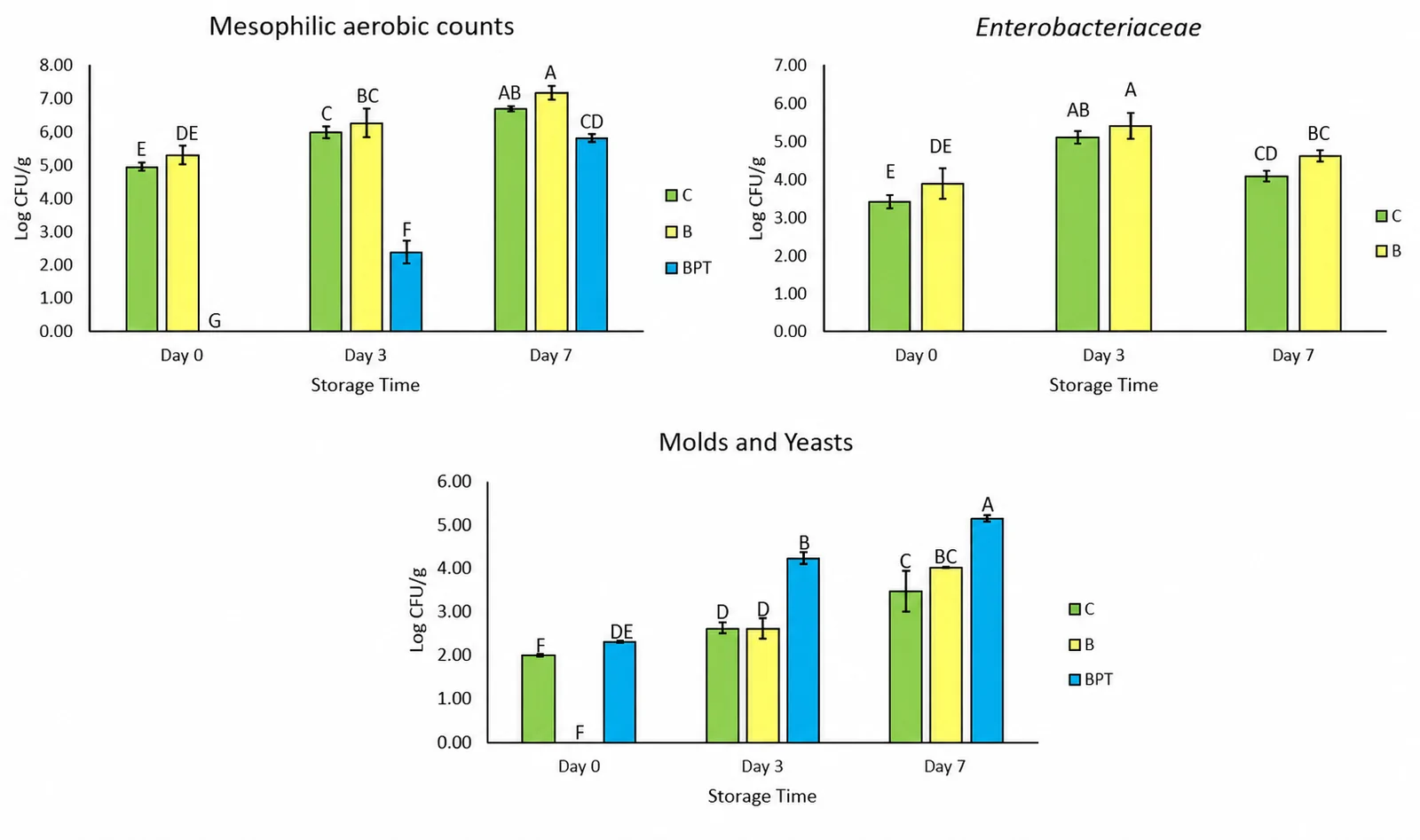
Microbiological counts of the eye of round beef during storage. Different uppercase letters (A–G) indicate significant differences (*p* < 0.05). C: Unmarinated samples; B: Brine-marinated samples; BPT: Tamarind-brine marinated samples. Error bars represent the standard error of the mean.

All microbiological counts were significantly affected by treatment, storage time, and their interaction (*p* < 0.05) (Figure 2), indicating that both marinade composition and storage conditions influenced microbial dynamics throughout refrigerated storage. In the BPT treatment, mesophilic aerobic counts were below the detection limit (1 log CFU/g) at the beginning of storage, suggesting an initial suppression of bacterial growth. This response is likely associated with the low pH of tamarind pulp, combined with its high levels of organic acids and bioactive compounds, which are known to inhibit acid-sensitive bacterial populations. During storage, mesophilic aerobic counts increased (*p* < 0.05) in all treatments, probably reflecting the progressive proliferation of microorganisms capable of adapting to refrigerated and acidic conditions, including acid-tolerant bacteria such as lactic acid bacteria, although this microbial group was not specifically evaluated in the present study. Likewise, psychrotrophic microorganisms, which play an important role in the spoilage of refrigerated meat products, were not evaluated. Despite this, BPT exhibited the lowest mesophilic aerobic counts at the end of storage (5.80 log CFU/g), indicating that the tamarind-based marinade maintained a selective inhibitory effect against bacterial growth throughout the storage period.

*Enterobacteriaceae* remained below the detection limit (1 log CFU/g) in BPT samples throughout storage, whereas they were detected in the C and B treatments, reaching maximum counts on day 3 (5.18 and 5.35 log CFU/g, respectively). This behavior is consistent with the high sensitivity of *Enterobacteriaceae* to acidic environments and may also be associated with the presence of antimicrobial phytochemicals in tamarind pulp, including tannins and terpenoids [9,45,49]. In contrast, NaCl alone (B treatment) was insufficient to effectively limit the growth of this bacterial group under the conditions evaluated.

Regarding molds and yeasts, BPT showed significantly higher counts than the other treatments on days 3 and 7 (*p* < 0.05), indicating that these microorganisms were less affected by the acidic environment created by the tamarind-based marinade. Mold and yeast populations increased during storage in all treatments (*p* < 0.05), reflecting the progressive colonization of the product under refrigerated conditions. These findings indicate that tamarind pulp did not exert a broad-spectrum antimicrobial effect but rather a selective antimicrobial action, effectively suppressing acid-sensitive bacterial groups, particularly *Enterobacteriaceae*, while providing limited control over acid-tolerant spoilage microorganisms such as molds and yeasts. However, because lactic acid bacteria and psychrotrophic microorganisms were not evaluated, and mold and yeast populations increased during storage, the present results should not be interpreted as demonstrating an overall extension of microbiological shelf life. Finally, *Salmonella* spp. were not detected in any treatment throughout refrigerated storage, indicating that all samples complied with the microbiological safety standards established for the conditions evaluated.

### 3.9. Sensory Analysis

**Figure 3:**
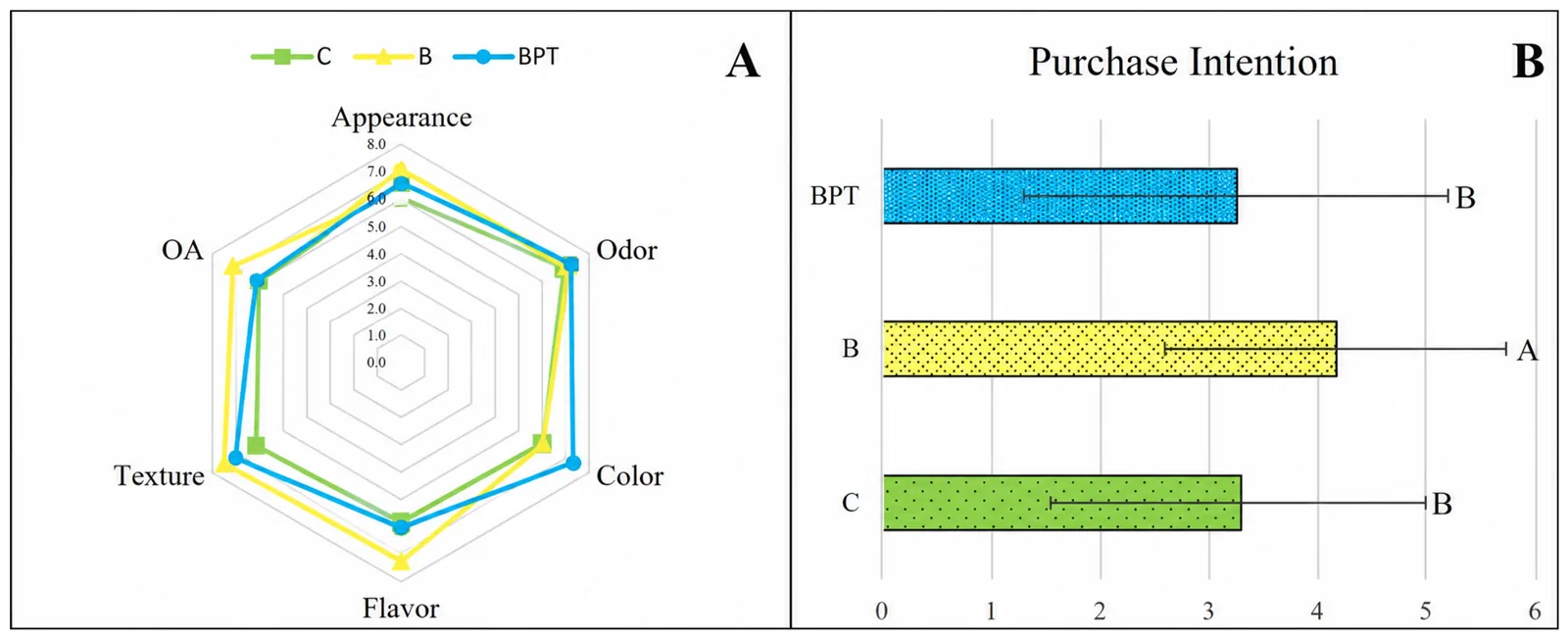
Sensory scores (**A**) and purchase intention (**B**) of eye of round beef. Different uppercase letters (A–B) indicate significant differences (*p* < 0.05). OA: Overall acceptability. C: Unmarinated samples; B: Brine-marinated samples; BPT: Tamarind-brine marinated samples.

The sensory evaluation of eye of round beef samples is presented in Figure 3. Overall liking scores (Figure 3A) indicated that treatments B and BPT significantly influenced most sensory attributes (*p* < 0.05), except for color. It means that despite differences in color parameters, the marination process did not negatively affect sensory liking or the purchasing decision. The B treatment received higher scores for flavor, texture, and overall acceptability, whereas the BPT treatment was more favorably rated for odor and appearance. The greater acceptance of B in these attributes may be associated with its more conventional sensory profile, characterized by a salt-enhanced taste and typical meaty flavor attributes. In contrast, the incorporation of tamarind pulp introduced distinct sensory notes, including acidic and fruity characteristics, that modified the traditional beef flavor profile and may have influenced consumer perception. This shift was also reflected in purchase intention (Figure 3B), where no significant difference was observed between C and BPT (*p* > 0.05), suggesting that the inclusion of tamarind did not enhance consumer willingness to purchase compared to the control.

The results of the CATA analysis (Table 4) confirmed that marination significantly influenced consumer perception. Attributes such as a shiny appearance, a salty taste, and a tender texture were more frequently associated with the B and BPT treatments (*p* < 0.05), aligning with instrumental findings, including higher L* values, increased salt content, and similar texture profiles at the initial stage of storage. In contrast, the BPT treatment was more frequently described as having a fruity taste, a fruity odor, an acidic odor, a sweet odor, a brownish color, and a bitter taste (*p* < 0.05), reflecting the intrinsic characteristics of tamarind pulp. These descriptors are consistent with physicochemical results, including lower pH and higher b*, indicating that these attributes directly influenced sensory perception.

**Figure 4:**
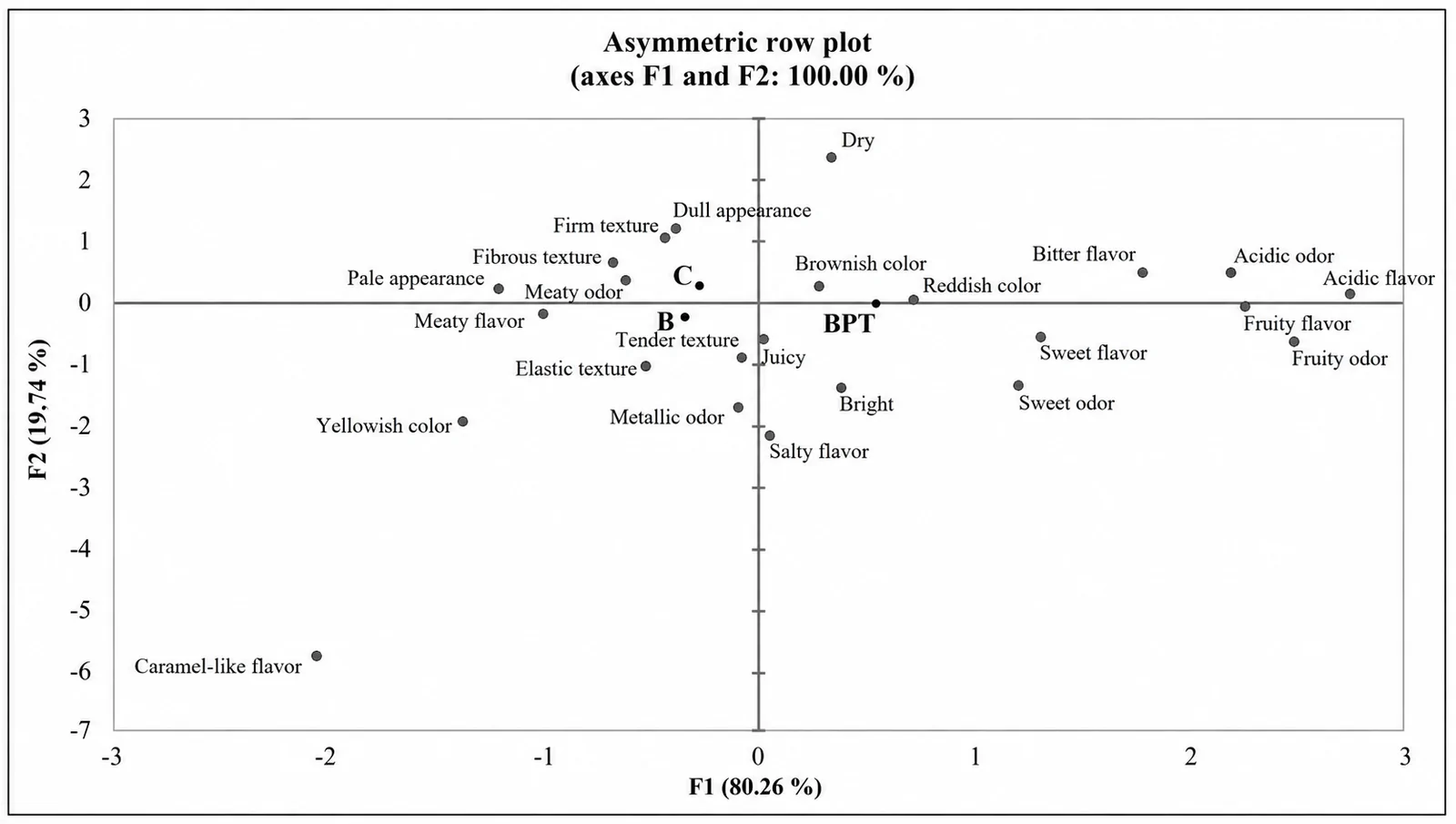
Correspondence analysis for CATA terms of eye of round beef. C: Unmarinated samples; B: Brine-marinated samples; BPT: Tamarind-brine marinated samples.

Correspondence analysis (Figure 4) further highlighted a distinct sensory profile among treatments. Samples from C and B were primarily associated with typical meat attributes, such as meaty flavor and odor, whereas BPT was clearly associated with fruity, acidic, and sweety flavors and odors. Additionally, attributes considered less desirable for meat quality, such as dry, fibrous, and firm texture and pale appearance, were more frequently associated with the C treatment, suggesting that marination with tamarind pulp improved certain sensory perceptions.

Overall, the incorporation of tamarind pulp resulted in a marked shift in the sensory profile of the eye of round beef, introducing novel flavor and aroma characteristics. While these changes did not enhance overall acceptance compared to B, they did not substantially compromise consumer perception and may appeal to specific consumer segments seeking differentiated or more complex flavor profiles.

## 4. Conclusions

The present study demonstrated that tamarind pulp-based marination substantially influenced the physicochemical quality, oxidative stability, microbiological characteristics, and sensory attributes of eye of round beef. Compared with conventional brine marination, the incorporation of tamarind pulp reduced pH and WHC, resulting in changes in proximate composition, instrumental color, and texture. Although lower hardness and chewiness were observed throughout storage, these technological modifications were accompanied by reduced water retention and pronounced color changes.

From a microbiological and oxidative perspective, tamarind pulp selectively affected product stability by suppressing *Enterobacteriaceae* and reducing lipid oxidation compared with brine-marinated samples. However, these effects did not extend uniformly to all microbial groups, as molds and yeasts exhibited higher counts during storage, emphasizing the selective nature of the antimicrobial action of tamarind pulp. Consequently, the microbiological findings should be interpreted as evidence of selective inhibition of specific bacterial groups rather than an overall improvement in microbiological shelf life.

Tamarind incorporation also produced a distinctive sensory profile characterized by fruity, acidic, and sweet notes. Nevertheless, although these sensory modifications differentiated the product from conventionally marinated beef, they did not improve overall acceptability relative to the brine treatment, which received the highest scores for flavor and overall acceptance.

Overall, tamarind pulp represents a promising natural ingredient capable of modulating multiple quality attributes of marinated eye of round beef. These findings provide initial experimental evidence supporting further investigation of tamarind pulp as a natural ingredient for clean-label meat products. However, additional studies addressing ingredient standardization, comprehensive microbiological stability, sensory acceptance across different consumer populations, and industrial-scale processing are necessary before broader commercial application can be recommended. Therefore, the application of tamarind pulp in clean-label meat products should consider both advantages and undesirable effects, and further optimization of formulation and processing conditions is needed to maximize its technological and microbiological benefits.

## Figures and Tables

**Table 1:** Technological properties (%) and proximate composition (%) of eye of round beef.

	Treatments					
Parameters	C	B	BPT	Overall Means	SEM	Significance
MLU	−1.66 ^C^	9.27 ^A^	2.86 ^B^	3.50	0.18	***
WHC	84.26 ^A^	82.33 ^A^	74.16 ^B^	80.25	1.21	**
CL	34.36 ^A^	37.66 ^A^	36.97 ^A^	36.30	0.54	n.s.
Moisture	71.26 ^B^	71.32 ^A^	65.65 ^C^	69.41	0.05	***
Protein	23.96 ^A^	18.97 ^C^	22.39 ^B^	21.77	0.07	***
Fat	3.59 ^C^	6.54 ^B^	7.22 ^A^	5.78	0.06	***
Ash	1.99 ^C^	2.32 ^B^	2.45 ^A^	2.25	0.04	***
Carbohydrates	0.00 ^C^	0.85 ^B^	2.31 ^A^	1.05	0.01	***
Salt content	0.09 ^B^	1.02 ^A^	1.04 ^A^	0.72	0.01	***

Different uppercase superscripts (A–C) on the same row indicate significant differences (*p* < 0.05). SEM: Standard error of the mean. MLU: Marinate liquid uptake; WHC: Water-holding capacity; CL: Cooking loss; C: Unmarinated samples; B: Brine-marinated samples; BPT: Tamarind-brine marinated samples; **: *p* < 0.01; ***: *p* < 0.001; n.s.: not significant.

**Table 2:** Water activity (a_w_), pH, and TBARS (mg MDA/kg) of eye of round beef during storage.

		Treatments					Significance		
	Day	C	B	BPT	Means (S)	SEM	T	S	T × S
a_w_	0	0.979 ^AB^	0.979 ^AB^	0.974 ^BC^	0.977	0.003	***	*	**
	3	0.982 ^A^	0.980 ^A^	0.969 ^C^	0.977	0.005			
	7	0.981 ^A^	0.979 ^A^	0.977 ^AB^	0.979	0.001			
	Means (T)	0.980	0.979	0.973					
	SEM	0.002	0	0.003					
pH	0	5.46 ^AB^	5.53 ^A^	4.24 ^D^	5.07	0.63	***	***	n.s.
	3	5.42 ^B^	5.48 ^AB^	4.07 ^E^	4.99	0.69			
	7	5.27 ^C^	5.35 ^C^	4.02 ^E^	4.88	0.64			
	Means (T)	5.38	5.45	4.10					
	SEM	0.09	0.08	0.11					
TBARS	0	0.145 ^CDE^	0.220 ^AB^	0.241 ^A^	0.202	0.049	***	**	*
	3	0.150 ^BCDE^	0.225 ^A^	0.199 ^ABCD^	0.191	0.042			
	7	0.119 ^E^	0.213 ^ABC^	0.133 ^DE^	0.155	0.046			
	Means (T)	0.138	0.219	0.191					
	SEM	0.029	0.019	0.051					

Different uppercase superscripts (A–E) indicate significant differences (*p* < 0.05). SEM: Standard error of the mean. C: Unmarinated samples; B: Brine-marinated samples; BPT: Tamarind-brine marinated samples. T: treatments (C, B, BPT); S: storage time (0, 3, 7 days); *: *p* < 0.05; **: *p* < 0.01; ***: *p* < 0.001; n.s.: not significant.

**Table 3:** Color parameters of the eye of round beef during storage.

		Treatments					Significance		
	Day	C	B	BPT	Means (S)	SEM	T	S	T × S
L*	0	46.3 ^BC^	51.0 ^A^	52.2 ^A^	49.84	3.20	***	n.s	n.s
	3	43.8 ^BC^	49.6 ^AB^	52.3 ^A^	48.58	3.77			
	7	46.1 ^C^	48.9 ^AB^	51.6 ^A^	48.90	2.83			
	Means (T)	45.4	49.9	52.1					
	SEM	1.76	1.66	1.57					
a*	0	13.2 ^A^	6.4 ^C^	7.5 ^BC^	9.08	3.31	***	*	**
	3	13.9 ^A^	7.9 ^BC^	7.8 ^BC^	9.86	3.05			
	7	10.3 ^B^	8.6 ^BC^	6.3 ^C^	8.43	2.22			
	Means (T)	12.5	7.6	7.2					
	SEM	2.26	1.33	0.77					
b*	0	9.7 ^BC^	6.7 ^C^	14.7 ^A^	10.37	3.56	***	**	***
	3	8.8 ^BC^	8.0 ^BC^	16.3 ^A^	11.06	4.04			
	7	7.4 ^BC^	8.5 ^BC^	11.9 ^B^	9.29	2.13			
	Means (T)	8.6	7.8	14.3					
	SEM	1.26	0.94	2.29					
C*	0	16.4 ^AB^	9.3 ^D^	16.5 ^AB^	14.09	3.78	***	**	***
	3	16.5 ^AB^	11.2 ^CD^	18.1 ^A^	15.28	3.24			
	7	12.7 ^C^	12.1 ^CD^	13.5 ^BC^	12.80	1.52			
	Means (T)	15.2	10.9	16.0					
	SEM	2.45	1.55	2.34					
*h*°	0	36.3 ^C^	46.1 ^B^	62.7 ^A^	48.41	11.53	***	n.s.	n.s.
	3	32.3 ^C^	45.5 ^B^	64.5 ^A^	47.43	13.85			
	7	35.9 ^C^	45.1 ^B^	61.9 ^A^	47.64	11.79			
	Means (T)	34.8	45.6	63.0					
	SEM	3.31	2.49	2.13					

Different uppercase superscripts (A–D) indicate significant differences (*p* < 0.05). SEM: Standard error of the mean. C: Unmarinated samples; B: Brine-marinated samples; BPT: Tamarind-brine marinated samples. T: treatments (C, B, BPT); S: storage time (0, 3, 7 days); *: *p* < 0.05; **: *p* < 0.01; ***: *p* < 0.001; n.s.: not significant.

**Table 4:** Check-All-That-Apply (CATA) terms for eye of beef round.

	Treatments				
Terms	C	B	BPT	SEM	Significance
Shiny	7 ^B^	17 ^A^	19 ^A^	0.68	*
Fibrous texture	24 ^A^	21 ^A^	13 ^A^	-	n.s.
Juicy	21 ^A^	31 ^A^	30 ^A^	-	n.s.
Caramel-like flavor	0 ^A^	2 ^A^	0 ^A^	-	n.s.
Dry	28 ^A^	7 ^B^	24 ^A^	0.71	***
Fruity odor	1 ^B^	2 ^B^	16 ^A^	0.05	***
Salty taste	7 ^B^	27 ^A^	21 ^A^	0.07	***
Metallic odor	3 ^A^	8 ^A^	6 ^A^	-	n.s.
Fruity taste	4 ^B^	3 ^B^	27 ^A^	0.06	***
Meaty flavor	47 ^A^	58 ^A^	22 ^B^	0.09	***
Elastic texture	9 ^A^	16 ^A^	9 ^A^	-	n.s.
Reddish color	1 ^A^	1 ^A^	2 ^A^	-	n.s.
Brownish color	44 ^B^	41 ^B^	59 ^A^	0.07	**
Firm texture	25 ^A^	18 ^A^	16 ^A^	-	n.s.
Sweet taste	2 ^A^	3 ^A^	8 ^A^	-	n.s.
Acidic odor	3 ^B^	1 ^B^	14 ^A^	0.05	***
Dull appearance	42 ^A^	28 ^B^	27 ^B^	0.08	*
Meaty odor	49 ^A^	48 ^A^	30 ^B^	0.08	***
Acidic taste	5 ^B^	1 ^B^	47 ^A^	0.08	***
Yellowish color	5 ^AB^	12 ^A^	2 ^B^	0.05	**
Tender texture	20 ^B^	34 ^A^	29 ^AB^	0.08	**
Bitter taste	2 ^AB^	1 ^B^	7 ^A^	0.03	**
Pale appearance	27 ^A^	29 ^A^	8 ^B^	0.08	***
Sweet odor	1 ^A^	3 ^A^	6 ^A^	-	n.s.
Shiny	7 ^B^	17 ^A^	19 ^A^	0.68	*

Different uppercase superscripts (A–B) in the same row indicate significant differences (*p* < 0.05). SEM: Standard error of the mean. C: Unmarinated samples; B: Brine-marinated samples; BPT: Tamarind-brine marinated samples. *: *p* < 0.05; **: *p* < 0.01; ***: *p* < 0.001; n.s.: not significant.

## Data Availability

The original contributions presented in the study are included in the article. Further inquiries can be directed to the corresponding authors.
